# Obese Subjects With Specific Gustatory Papillae Microbiota and Salivary Cues Display an Impairment to Sense Lipids

**DOI:** 10.1038/s41598-018-24619-1

**Published:** 2018-04-30

**Authors:** Philippe Besnard, Jeffrey E. Christensen, Hélène Brignot, Arnaud Bernard, Patricia Passilly-Degrace, Sophie Nicklaus, Jean-Paul Pais de Barros, Xavier Collet, Benjamin Lelouvier, Florence Servant, Vincent Blasco-Baque, Bruno Verges, Laurent Lagrost, Gilles Feron, Rémy Burcelin

**Affiliations:** 1 UMR Lipides/Nutrition/Cancer U1231 INSERM/Univ Bourgogne-Franche Comté/AgroSupDijon, 21000 Dijon, France; 2 I2MC Institut des maladies métaboliques et cardiovasculaires/UMR 1048 INSERM/Univ Toulouse III Paul Sabatier, 31400 Toulouse, France; 3 Centre des Sciences du Goût et de l’Alimentation, AgroSup Dijon, CNRS, INRA, Univ. Bourgogne Franche-Comté, F-21000 Dijon, France; 4 Vaiomer S.A.S, 31670 Labège, France

## Abstract

Some obese subjects overeat lipid-rich foods. The origin of this eating behavior is unknown. We have here tested the hypothesis that these subjects could be characterized by an impaired fatty taste sensitivity linked to a change in the gustatory papillae microbial and salivary environment. The composition of microbiota and saliva surrounding the circumvallate papillae was analyzed in combination with the orosensory lipid detection threshold in normal weight (NW) and obese (O) adults. Microbial architecture was similar to what was known in feces, but with an increased frequency of Proteobacteria. No difference in the orosensory sensitivity to lipids and composition of oral microbiota and saliva was observed between NW and O subjects. By contrast, specific bacterial and salivary signatures were found in lipid non-tasters, irrespectively of BMI. A multivariate approach highlighted that the salivary flow, lysozyme activity, total antioxidant capacity and TM7 bacterial family discriminated between tasters and non-tasters. Subgroup analysis of obese tasters (OT) versus obese non-tasters (ONT) identified specific bacterial metabolic pathways (*i.e*. phosphotransferase and simple sugar transport systems) as being higher in ONT. Altogether with the identification of a set of significant salivary variables, our study suggests that an “obese tongue” phenotype is associated with decreased orosensory sensitivity to lipids in some obese subjects.

## Introduction

The rapid progression of obesity worldwide constitutes a major public health challenge by reason of deleterious effects of associated pathologies (type-2 diabetes, cardiovascular diseases, strokes, hypertension, cancers, and neurodegenerative diseases) to cite a few. Recent changes in our lifestyle, especially the easy access to low cost, energy-dense foods coupled with a decreased physical activity contributes significantly to this obesity epidemic. Importantly, the sensory appeal for highly palatable foods rich in fat seems to be accentuated in some obese subjects^1–3^. The origin of this preferential food selection remains elusive. Nevertheless, an obesity-related dysfunction of the orosensory system responsible for the oral detection and central perception of dietary lipids may be suspected^4^. Consistent with this assumption, neuroimaging studies have highlighted the existence of structural remodeling and functional alterations in brain areas (*i.e*. cortico-mesolimbic system) involved both in food perception and reward processing in some obese subjects^5^. This “obese brain” phenotype^6^ might contribute to the susceptibility to overeat lipid-rich foods^7–9^. Paradoxically, association between obesity and alterations of oral parameters putatively involved in the orosensory fat detection by the gustatory papillae remains poorly investigated in humans. However, variations in the composition of oral microbiota might also impair the orosensory detection of dietary lipids since obesity is associated with a dysbiosis at the intestinal level^10,11^. Whether changes in the microbial ecology also take place in the oral cavity of obese subjects is unknown, but has been suggested. Differences in the composition of bacteria in saliva have been shown in overweight women as compared to NW subjects^12^ and an association between microbiota found in subgingival biofilms and obesity has been reported in adolescents^13^. Unfortunately, these data remain poorly predictive of a putative influence of oral microbiota on the orosensory detection of fat since oral microbiome is greatly heterogenous^14^. Indeed, oral cavity encompasses multiple ecological niches (*i.e*. tongue surfaces, gingival sulcus, teeth, lip, soft and hard palate, saliva) colonized by distinct microbial communities displaying specific features (*i.e*. diversity and stability/variability – see Human Oral Microbiome Database, www.homd.org) influenced by local challenges (*i.e*. oxygen variation, nutrient availability, mechanical stress, salivary flow and composition)^15^. Papillary structure of the dorsal tongue constitutes the major microbiota reservoir of the oral cavity, bacterial load increasing along an anteroposterior axis^16^. The dorsal surface of the tongue is mainly covered with tactile filiform papillae between which three types of gustatory papillae (*i.e., fungiforms, foliates and circumvallates*) are distributed according different spatial locations. The most of the taste buds are found in the dozen circumvallate papillae (CVP) located in the posterior part of the tongue. CVP are characterized by a dome-shape structure with a circular depression connected to the von Ebner’s glands^17^ known to produce salivary enzymes including lipases^18^. Such an anatomical layout might constitute an ecological niche for specific bacterial communities. Indeed, the composition of oral microbiota is highly sensitive to changes in environmental conditions, especially to variations in the salivary flux and composition (*i.e*. pH, redox potential, enzymatic activities)^14^. Interestingly, obesity seems to be associated with modifications in salivary parameters known to play a significant role in the oral fat detection, such as flux of saliva or lipase activity^19^. However, whether specific features of the microbiota and/or saliva surrounding the CVP contribute to obesity by compromising orosensory sensitivity to lipids remains to be determined. In accordance with these observations, we hypothesized that obesity might also be characterized in some subjects by an “obese tongue” phenotype leading to a change in sensitivity of their oral fat detection system. Therefore, the orosensory detection threshold of a fatty acid widely found in foods (*i.e*. linoleic acid, LA), microbiota composition in the direct vicinity of CVP, and salivary parameters known to be involved in the oral fat sensitivity were determined in NW (BMI < 25 kg/m^2^) and O (BMI ≥ 30 kg/m^2^) volunteers. To establish which physiological, microbiotal, and/or salivary variables were the most discriminant to characterize these subjects, we performed three complementary comparisons on: (i) BMI (NW *vs* O), (ii) sensitivity of oral fat detection in all subjects (LA taster [T] *vs* LA non-taster [NT]) and (iii) in obese only (obese taster [OT] *vs* obese non-taster [ONT]).

## Results

### Orosensory sensitivity to LA

**Figure 1:**
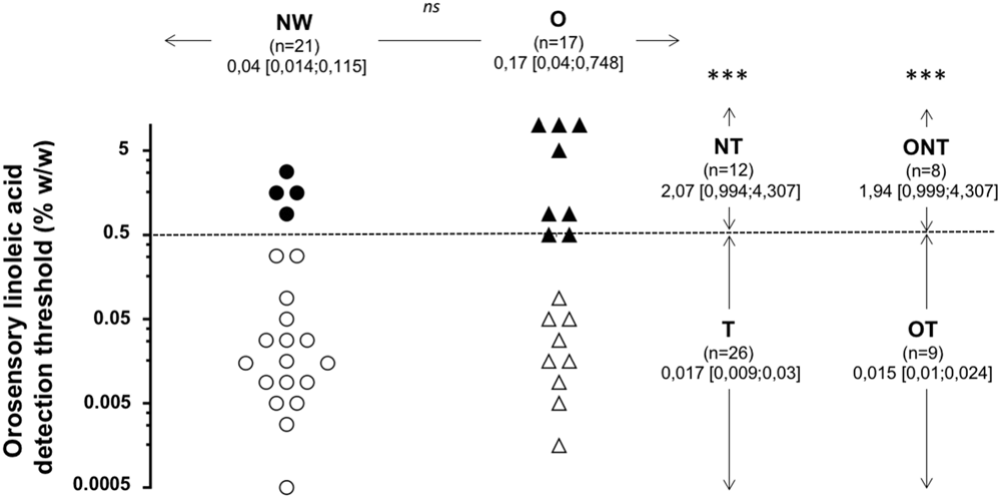
Orosensory detection threshold of linoleic acid (LA) determined by using the 3-alternative forced-choice (3-AFC) ascending concentration procedure in normal weight (NW, BMI < 25 kg/m^2^) and obese (O, BMI ≥ 30 kg/m^2^) subjects. 3-AFC was carried out on emulsions in an ascending concentration from 0.00028% to 5% LA (wt/wt) spaced by 0.25 log units (18 solutions in total). Data were analyzed according three complementary comparisons: NW *vs* O, LA taster (T) *vs* LA non-taster (NT) and obese taster (OT) *vs* obese non-taster (ONT). To characterize the subjects in function of their oral LA sensitivity, it was arbitrary postulated that individuals displaying a threshold value ≥ 0.5% LA were non-tasters (NT). Geometric means; ns, non-significant, ***p < 0;001.

The orosensory perception of dietary lipids is a multimodal phenomenon involving gustatory, olfactory and textural sensations. To minimize olfactory and textural inputs, experiments were conducted in subjects wearing a nose clip and using a textured solution leading to similar particle size regardless of the LA concentration being tested^20,21^. An important inter-individual variability was found with the distribution of LA detection threshold covering five orders of magnitude both in NW and O groups (Fig. 1). Therefore, no significant difference in LA detection threshold was found between the two groups. To further characterize these subjects, it was arbitrarily postulated that individuals displaying a threshold value ≥ 0.5% LA were NT. Although T and NT subjects were found both in NW and O groups, this new analysis has led to discriminant data (P < 0.001). Nevertheless, ONT were more numerous than NWNT (n = 8 and 4, respectively - Fig. 1), suggesting that obesity increases the susceptibility to be poorly sensitive to oral lipid stimulation. Interestingly, a clear difference between OT and ONT was also found, confirming that LA detection threshold is not strictly dependent on BMI^22^. Therefore, the microbiota and salivary data were next analyzed according to three complementary comparisons: NW *vs* O, T *vs* NT and OT *vs* ONT (Fig. 1).

### Microbiota ecology surrounding circumvallate papillae and body weight

**Figure 2:**
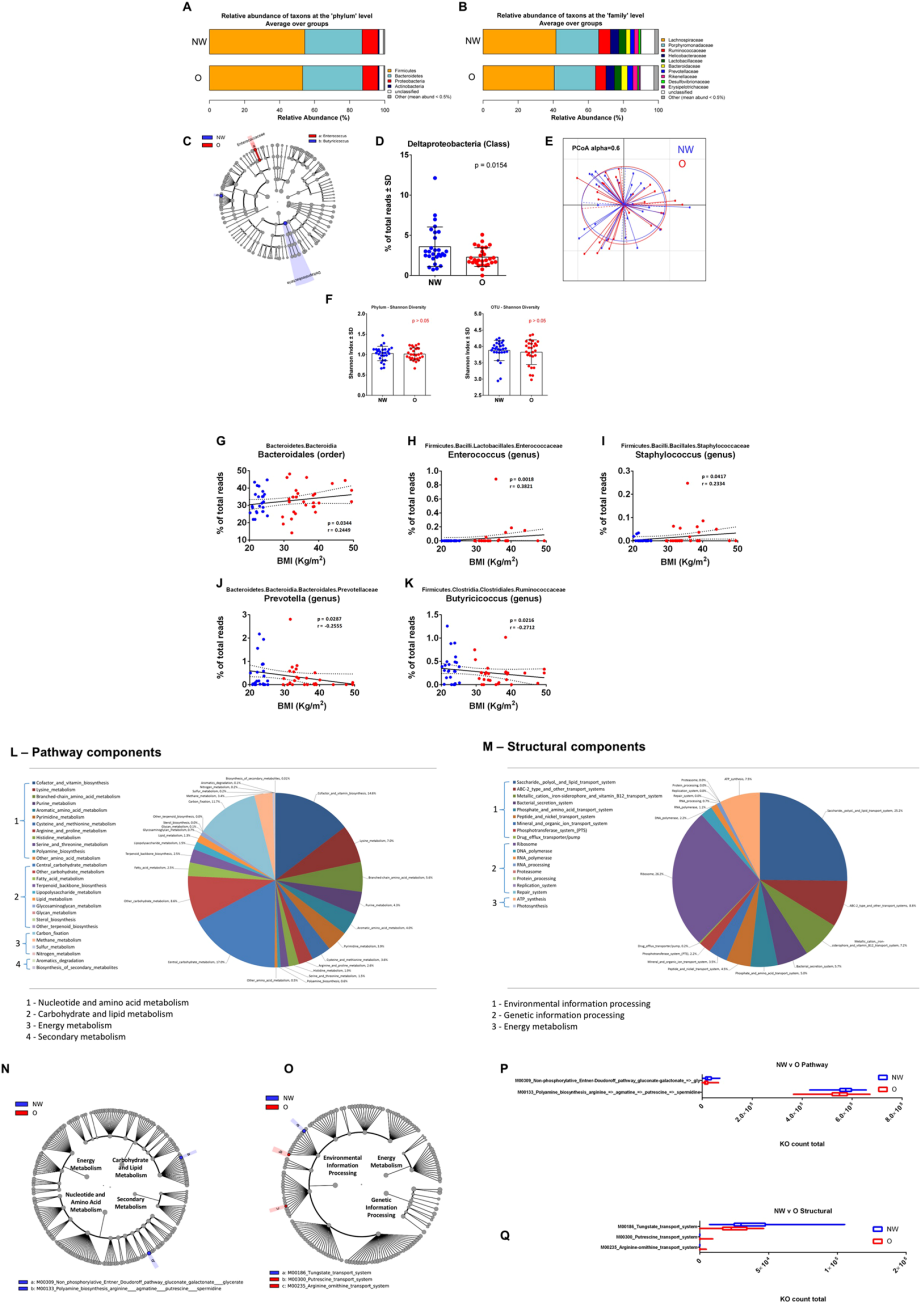
Relative abundance, alpha diversity, and dimensional reduction plot of CPV content microbiomes from NW vs O subjects. (**A**,**B**) Stacked column bar graphs depicting the different group average relative abundances and distribution of the most highly abundant resolved taxa at the (**A**) phylum and (**B**) family level for this study. (**C**) Cladograms derived from pairwise group LEfSe analysis of 16S sequences from CPV contents in NW vs O subjects. The cladograms show the taxonomic levels represented by rings with phyla at the innermost ring and genera at the outermost ring, and each circle is a member within that level. Taxa at each level are shaded (blue or red) according to the phenotype in which it is more abundant (P < 0.05; LDA score 2.0). (**D**) Bar graph analysis of the frequency of Deltaproteobacteria in both groups of subjects, significance is indicated. (**E**) Principal Component Orthogonal Analysis of the microbiota profile in both groups. The alpha cutoff is mentioned. (**F**) Plot of microbiome alpha diversity (Shannon index) for each CPV content sample at the phylum and family taxonomic levels. (**G**–**K**) Linear regression analyses between the frequency of the mentioned bacterial taxa and BMI. Significance, and linear regression coefficient are indicated on each panel. (**L**,**M**) Pathway component and structural components derived from pairwise group analysis of predicted metagenomics analysis (PICRUSt) of 16S sequences from CPV contents in NW vs O subjects. Major metabolic pathways are mentioned. (**N**,**O**) LDA Effect Size (LEfSe) cladograms of KEGG pathway contributions of predicted metagenomic data for CPV samples. Pairwise analysis was performed for NW vs O subjects. The cladograms show the KEGG pathway hierarchy represented by rings with the consolidated pathway modules (identified in the legend) at the outermost ring, and each circle is a member within that level. KEGG modules are shaded in color according to the group in which it is most abundant (P < 0.05; LDA score 2.0). The respective KEGG pathway cladogram legends are incorporated into the graphical representation (**P**,**Q**) of the relative predicted gene count for each differential feature.

To delineate for the first time the gustatory papillae microbiota ecology and whether this ecology is related to the subjects, body weight, swab samples were taken directly from the V-shaped row of the circumvallate papillae at the back of the tongue dorsa to collect the CVP associated microbiota. The CVP samples were extracted and sequenced to obtain the16S rRNA gene profiles for NW and O subjects (n = 45). Similar to what has been found in feces, the CVP microbiome was found to be mainly composed of anaerobic Firmicutes and Bacteroidetes phyla (Fig. 2A). Interestingly, the frequency of Proteobacteria, which reached ~10%, was higher than what was previously found in fecal samples. At the family taxonomic level, we observed a reduction of the frequency of Porphyromonadaceae in favor of a doubling of the Bacteroidaceae in the O group (Fig. 2B). To more precisely identify the differential taxa at all taxonomic levels we performed linear discriminant analysis^23^ effect size (LEfSe; http://huttenhower.sph.harvard.edu/gal) with the 16S rRNA sequence data^24^ (Fig. 2C). Only the abundance of the Deltaproteobacteria was slightly lowered in the O subjects (Fig. 2D). The primary coordinate analyses confirmed that the two ecologies were similar (Fig. 2E). We further quantified whether there were changes in the alpha diversity and found no difference at any taxonomic levels (Fig. 2F). Therefore, we performed linear regression to identify the taxa that could be associated with the BMI as a continuous variable. We identified that the Bacteroidales (order), *Enterococcus* (genus), and *Staphylococcus* (genus) were positively correlated with the BMI (Fig. 2G–I). In contrast, we found *Prevotella* (genus) and *Butyricicoccus* (genus) to be negatively correlated with BMI (Fig. 2J,K).

To gain insight into the potential molecular functions of the CVP associated bacterial microbiome, we used PICRUSt to predict the metagenomic contributions^25^. PICRUSt predicts potential metagenomes by ascribing annotated genes within a known sequenced database, such as the Kyoto Encyclopedia of Genes and Genomes (KEGG), with respect to the presence or absence of OTUs in 16S rRNA data sets. The predictive confidence can be assessed in PICRUSt by calculating the nearest sequenced taxon index (NSTI). The NSTI calculation quantifies the average branch length that separates each OTU from the closest sequenced reference bacterial genome with weighting for the abundance of that OTU in the sample. When NSTI number is low, it suggests that PICRUSt is likely to perform well in predicting the metagenome of the organisms in a sample. In this study, we used the KEGG database output for the reference OTUs and calculated a mean NSTI of 0.17 ± 0.02 s.d., which is considered a mid-range quality value and reported to produce accurate PICRUSt metagenome predictions^25^.

The composite predicted metagenomes were parsed into KEGG derived modules for pathway and structural complex prediction. Pathway modules represent functional units in KEGG metabolic pathway maps and structural complexes often form molecular machineries. For the overall cohort the data show that counts for pathway modules of genes encoding proteins involved in central carbohydrate metabolism, cofactor and vitamin biosynthesis, carbon fixation and other carbohydrate metabolism represent more than 50% of the pathways (Fig. 2L). For structural complex prediction, other than ribosomes, the data showed that counts for modules of genes encoding proteins involved in saccharide/polyol/lipid and ABC-2 type transport systems, ATP synthesis, cation/iron-siderophore/vitamin B12 transport systems, and bacterial secretion systems accounted for more than 50% of the predicted genes (Fig. 2M). LEfSe was then used to analyze the pathway and structural complex data for predicted gene enrichment using the same group comparisons as for the taxonomic evaluations (Fig. 2N,O). Only minor differences were identified between the cumulative predicted metagenomes of NW and O subjects, such as the non-phosphorylative Entner Doudoroff pathway and modules related to polyamine biosynthesis and arginine/putrescine transport systems (Fig. 2P,Q) (Mann–Whitney *P* < 0.05). Therefore, the taxonomic differences identified from CVP microbiomes from NW and O subjects have little impact on the predicted metabolic pathways. In brief, although a potential signature of variation in BMI was identified on the basis of the bacterial taxa surrounding CVP, it does not clearly discriminate the NW from O phenotypes and is not predictive of the oral sensitivity to lipids.

### Microbiota ecology surrounding circumvallate papillae and lipid tasting

**Figure 3:**
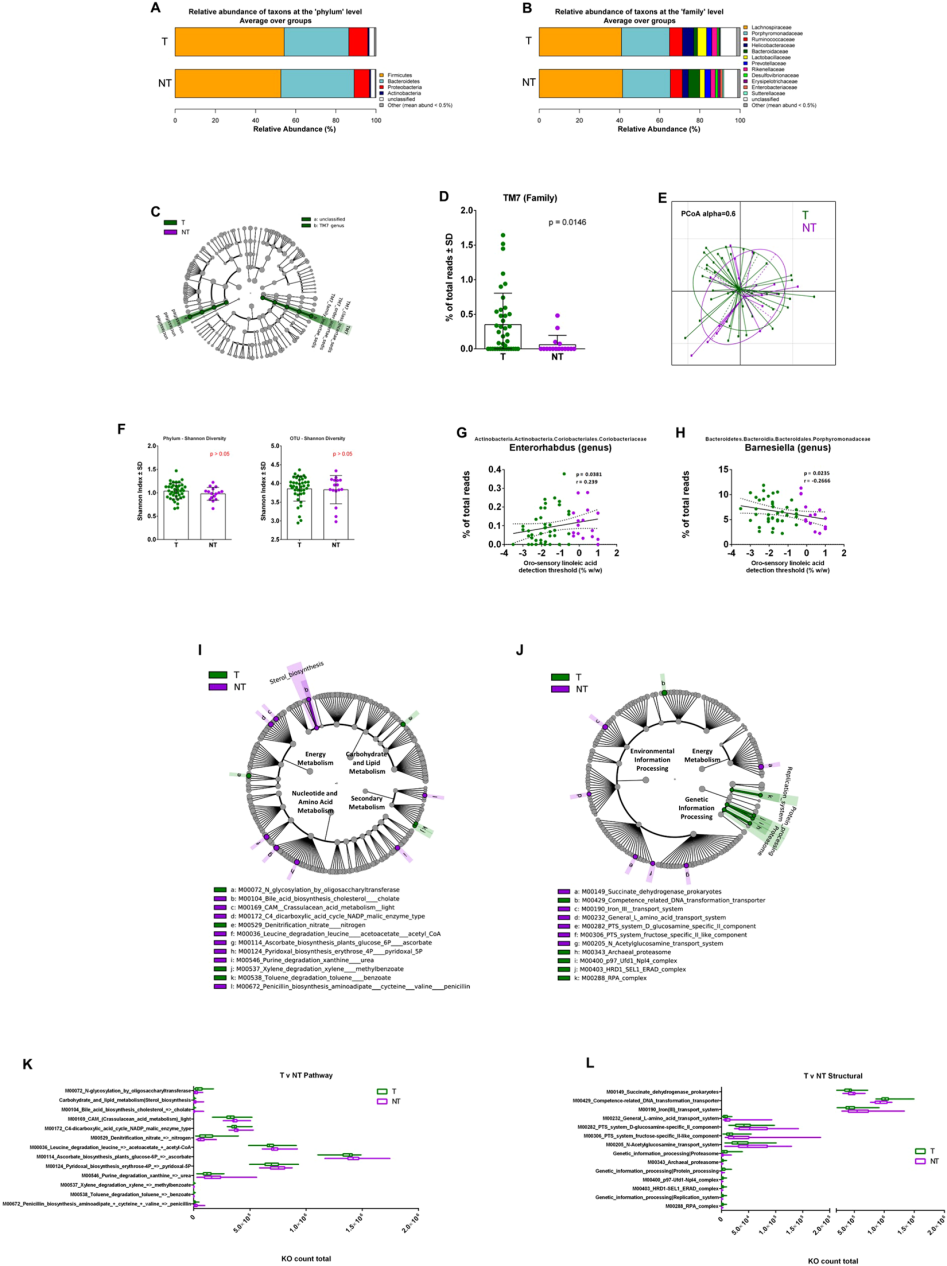
Relative abundance, alpha diversity, and dimensional reduction plot of CPV content microbiomes from T and NT subjects. (**A**,**B**) Stacked column bar graphs depicting the different group average relative abundances and distribution of the most highly abundant resolved taxa at the (**A**) phylum and (**B**) family level for this study. (**C**) Cladograms derived from pairwise group LEfSe analysis of 16S sequences from CPV contents in T vs NT subjects. Taxa at each level are shaded (green or purple) according to the phenotype in which it is more abundant (P < 0.05; LDA score 2.0). (**D**) bar graph analysis of the frequency of TM7 in both groups of subjects, significance is indicated. (**E**) Principal Component Orthogonal Analysis of the microbiota profile in both groups. The alpha cutoff is mentioned. (**F**) Plot of microbiome alpha diversity (Shannon index) for each CPV content sample at the phylum and family taxonomic levels. (**G,H**) Linear regression analyses between the frequency of the mentioned bacterial taxa and detection threshold of linoleic acid. Significance, and linear regression coefficient are indicated on each panel. (**I**,**J**) LDA Effect Size (LEfSe) cladograms of KEGG pathway component and structural components derived from pairwise group analysis of predicted metagenomics analysis (PICRUSt) of 16S sequences from CPV contents in T vs NT subjects. Major metabolic pathways are mentioned. (**K**,**L**) of the relative predicted gene count for each differential feature.

To delineate whether the lipid tasting sensitivity could depend upon variations in the corresponding microbiota ecology, we analyzed T and NT individuals irrespective of their BMI. The relative abundance barplots suggest no major differences between the frequency of the taxon at the phylum level between T and NT subjects (Fig. 3A). Although the phyla level profiles of these groups could be characterized by discrete differences, they were not statistically discriminant. At the family level, NT subjects were characterized by a rise of Bacteroidaceae and reduction of Helicobacteraceae and Lactobacillaceae, associated with a greater taxonomic variation at the family level (Erysipelotrichaceae, Enterobacteriaceae, Sutterellaceae) (Fig. 3B). LEfSe analysis demonstrated higher abundance of the TM7 genus and an unclassified bacteria genus in the T group (Fig. 3C). The frequency among subjects showed that the TM7 family was significantly higher in T individuals and infrequently detected in NT subjects (Fig. 3D). The primary coordinate analysis showed some dissimilarity between the groups (Fig. 3E). However, no statistical difference in the alpha diversity (Shannon index) of microbial ecology was identified (Fig. 3F). Linear regression analyses identified the *Enterorhabdus* genus as positively correlated and the *Barnesiella* genus as negatively correlated with LA tasting threshold (Fig. 3G,H).

PICRUSt analysis of pathways showed the highest differential orthologue counts for nucleotide and amino acid metabolism between T and NT subjects. Modules for leucine degradation, ascorbate biosynthesis, and pyridoxal biosynthesis were each elevated for NT subjects (Fig. 3I,J). However, these discriminant pathway modules each differed by less than 10% in mean abundance (Fig. 3K). In contrast, lower abundance modules for sterol and bile acid biosynthesis showed >3.5 fold higher count in the NT group, while modules for toluene and xylene degradation were >2.5 fold more abundant in the T group (Fig. 3L). Altogether, T and NT subjects could be differentiated based on the presence and frequency of a small set of bacteria. These differences impacted a moderate number of predicted metagenomic pathways and structural modules. This lack of striking difference between LA taster and LA non-taster groups may be linked to the heterogeneity of the study group, since this comparison includes both lean and obese individuals.

### Microbiota ecology surrounding circumvallate papillae in OT and ONT subjects

**Figure 4:**
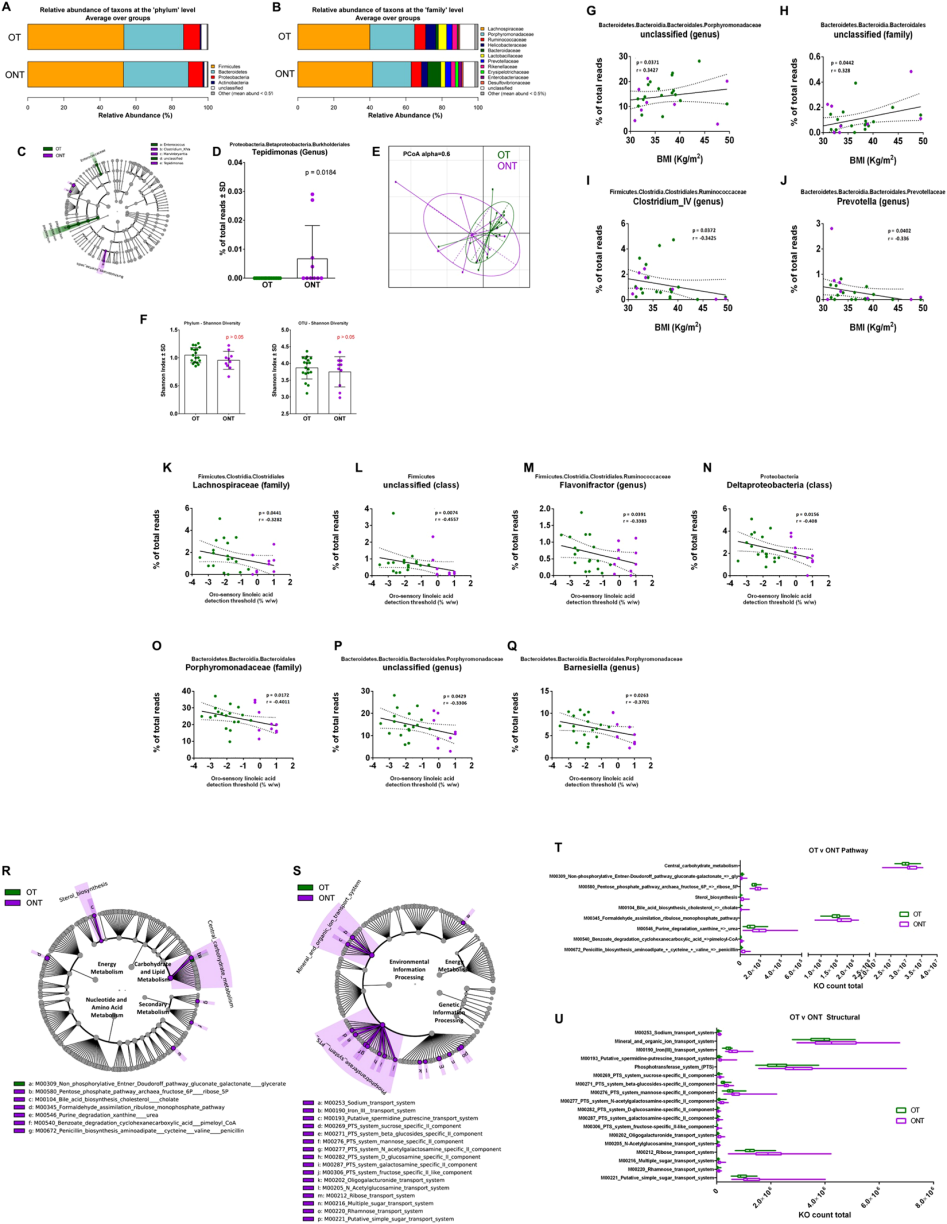
Relative abundance, alpha diversity, and dimensional reduction plot of CPV content microbiomes from OT and ONT subjects. (**A,B**) Stacked column bar graphs depicting the different group average relative abundances and distribution of the most highly abundant resolved taxa at the (**A**) phylum and (**B**) family level for this study. (**C**) Cladograms derived from pairwise group LEfSe analysis of 16S sequences from CPV contents in OT vs ONT subjects. Taxa at each level are shaded and colored according to the phenotype in which it is more abundant (P < 0.05; LDA score 2.0). (**D**) bar graph analysis of the frequency of Tepidimonas in both groups of subjects, significance is indicated. (**E**) Principal Component Orthogonal Analysis of the microbiota profile in both groups. The alpha cutoff is mentioned. (**F**) Plot of microbiome alpha diversity (Shannon index) for each CPV content sample at the phylum and family taxonomic levels. (**G**–**J**) linear regression analyses between the frequency of the mentioned bacterial taxa and BMI and (**K**–**Q**) with oro-sensory linoleic acid. Significance, and linear regression coefficient are indicated on each panel. (**R,S**) LDA Effect Size (LEfSe) cladograms of KEGG pathway component and structural components derived from pairwise group analysis of predicted metagenomics analysis (PICRUSt) of 16 S sequences from CPV contents in OT vs ONT subjects. Major metabolic pathways are mentioned. (**T,U**) of the relative predicted gene count for each differential feature.

To circumvent the issue of body weight heterogeneity in the T and NT subjects, we sought to determine the differences in frequencies of bacterial abundances for only the obese subjects in order to identify discriminative CVP taxonomic profiles. The presence of small differences in CVP microbiota relative abundances according to the body weight and lipid tasting sensitivity suggested that subgroups of these subjects could be identified. We therefore studied whether OT and ONT subjects were characterized by different microbiota ecologies. The barplot data show notably higher abundance of Bacteroidaceae and lower Lactobacillaceae and Helicobacteriaceae in the ONT subjects (Fig. 4A,B).

LEfSe analysis identified several taxonomic abundance differences between OT and ONT groups, with the most significant being the Burkholderiales (*Tepidomonas* genus) found to be increased in the ONT subjects (Fig. 4C,D). Principal coordinate analysis also showed greater dissimilarities between these two groups (Fig. 4E) than observed for the NW *vs* O and T *vs* NT comparisons. No significant difference was observed in the alpha diversity (Shannon index) at any taxonomic level, although there was a slightly lower mean value for ONT group (Fig. 4F). To explore whether bacterial taxa were correlated with the BMI in these obese subjects, linear regression analysis was performed. Two taxa were positively correlated with BMI (Porphyromonadaceae and an unclassified Bacteroidales family) and two taxa were negatively correlated with BMI (*Clostridium IV* and *Prevotella* genera) (Fig. 4G–J). We also used regression analysis to explore whether bacterial taxa were correlated with the LA tasting threshold in the obese subjects. Seven taxa were found to be negatively correlated with the LA tasting threshold (Fig. 4K–Q).

Consistent with differences in the CVP microbiomes between OT and ONT groups, we identified several differential pathway and structural features in the predicted metagenomes from PICRUSt analysis. ONT subjects were differentiated by an increased count in modules for metabolic pathways associated with central carbohydrate metabolism and bile acid modification (Fig. 4R,T). ONT subjects were also characterized by increased predicted structural components related to environmental information processing such as multiple phosphotransferase and simple sugar transport systems (Fig. 4S,U). Therefore, the microbiota surrounding CVP from OT and ONT subjects displays taxonomic and predicted metagenomic profiles which could assist explaining the LA tasting phenotype.

### LPS levels and lipid tasting

**Figure 5:**
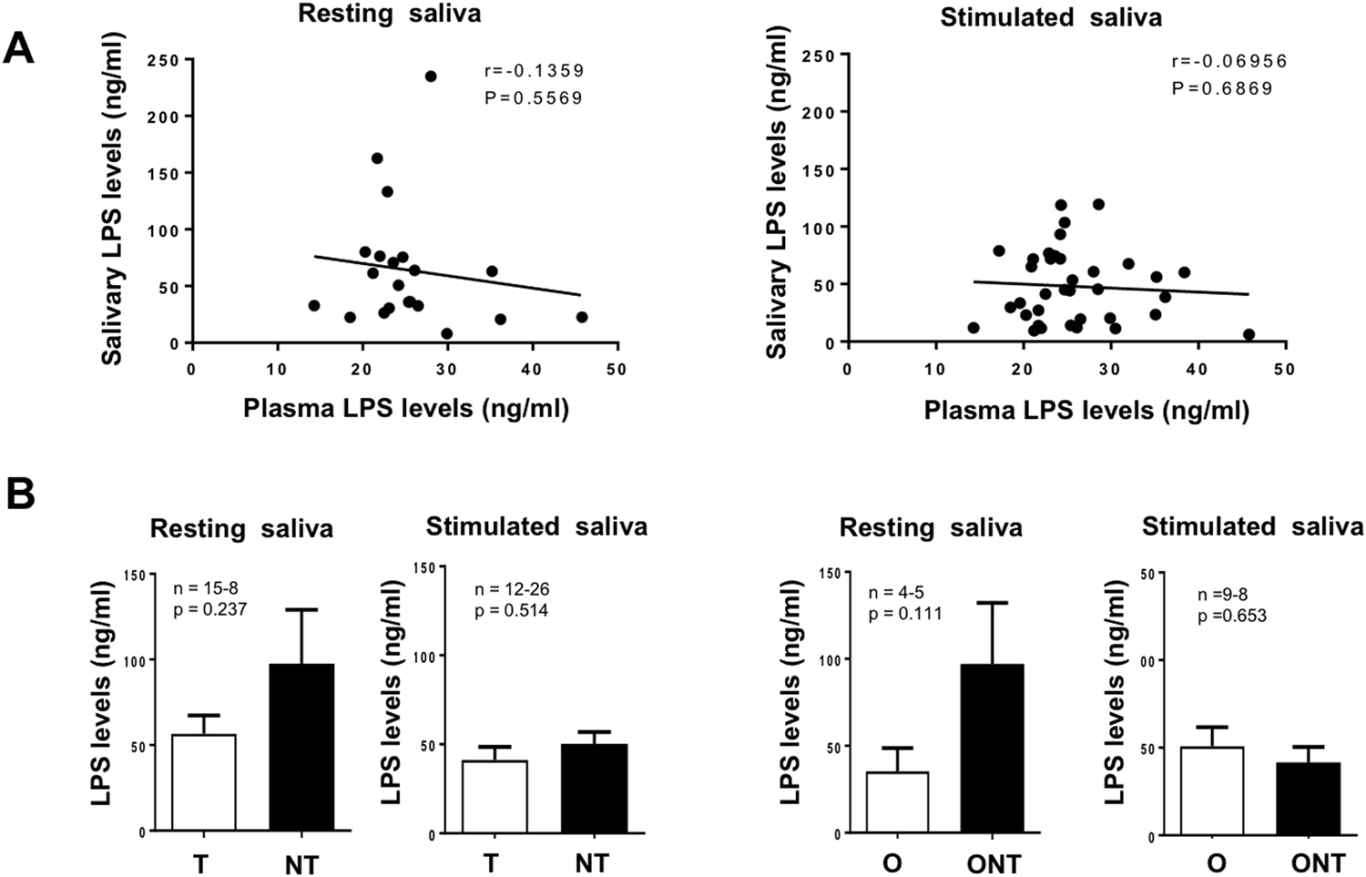
Analysis of lipopolyssaccharides (LPS) levels in blood and resting or stimulated saliva. (**A**) Correlation between blood and salivary LPS levels. (**B**) Comparison of salivary LPS levels in taster (T) and non-taster (NT) subjects. (**C**) Comparison of salivary LPS levels in obese taster (OT) and obese non-taster (ONT) subjects. Means ± SEM.

Since taste receptor cells are responsive to LPS stimulation^26,27^, LPS levels were assayed both in blood and saliva to explore the hypothesis of a possible relationship between fatty taste sensitivity and inflammatory state of gustatory papillae. As expected, blood LPS levels were higher in O than in NW subjects albeit a similar oral fat sensitivity. Amounts of LPS detected in blood and resting or stimulated saliva were not correlated demonstrating that salivary LPS levels were strictly dependent on the local microenvironment (Fig. 5A). Although the LPS levels in resting saliva tended to be higher in NT and ONT groups than in T and OT subjects, no significant difference was found (Fig. 5B,C). Moreover, this tendency was absent when the salivary flow was high, *i.e*. in stimulated saliva (Fig. 5B).

### Associations between selected biological parameters, salivary characteristics, microbiota composition and BMI or oral fat sensitivity

To further delineate the role of oral environment on the orosensory sensitivity to lipids, resting and stimulated saliva flux and composition were determined, and then were simultaneously analysed both with oral microbiota composition and a selection of biological determinants using a multivariate approach (MB-PLS-DA method) in NW *vs* O, T *vs* NT, and OT *vs* ONT groups. By merging data from different origins (blocks), this global statistical analysis is used to generate multi-variable predictive information on the functions of complex biological systems. Four blocks of predictors (*i.e*. selected biological determinants, resting saliva, stimulated saliva and microbiota composition – Table 1, supplemented data) were analyzed with regard to two components, body weight (NW *vs* O) and sensitivity of the orosensory lipid detection (T *vs* NT and OT *vs* ONT). Only projections of variables displaying a correlation coefficient >0.3 with a component were represented in figures. A variable importance in the projection (VIP) >1 highlighted parameters contributing significantly to the projection on the first component.

**Figure 6:**
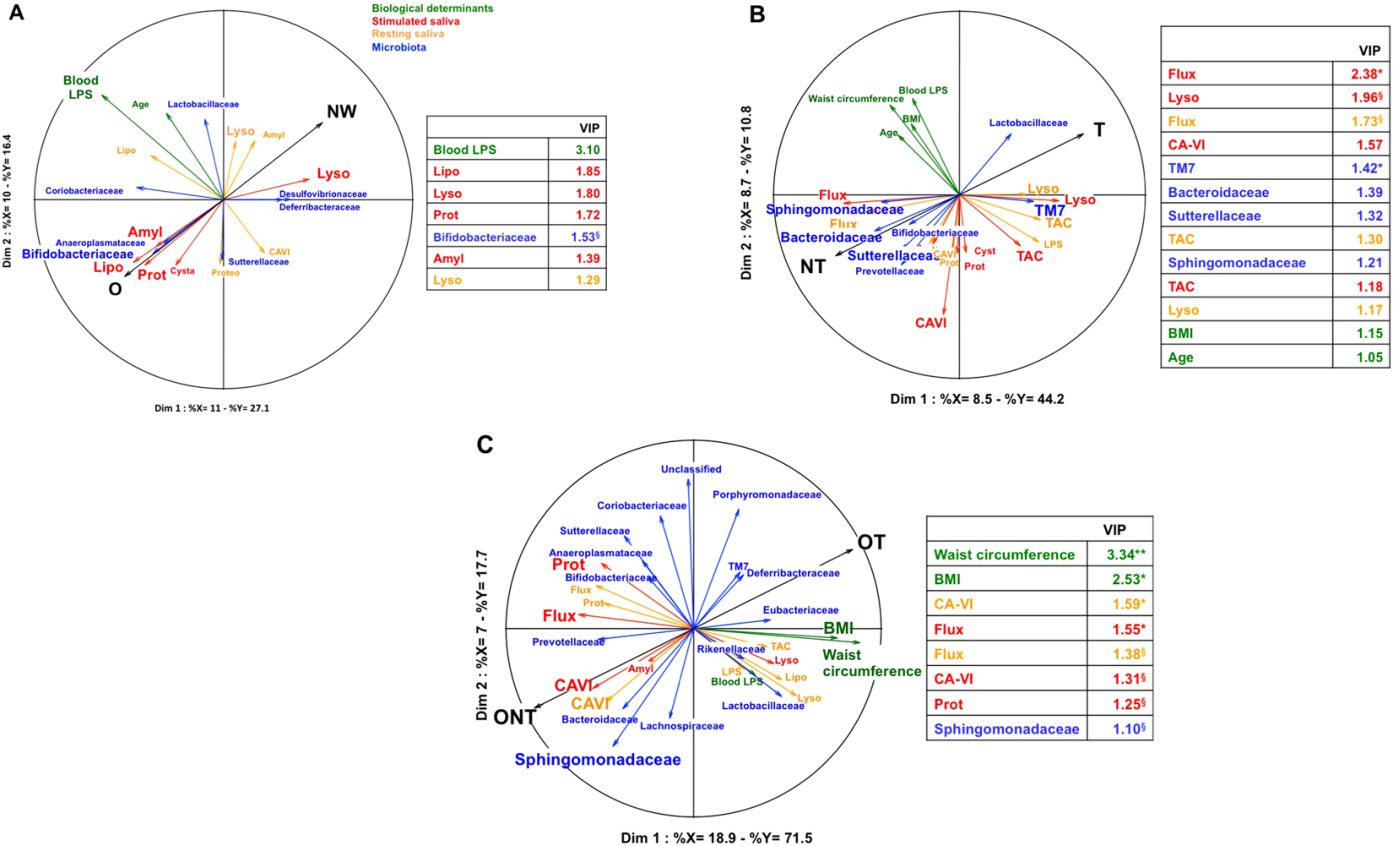
Partial Least Squares-Multiblocs-Discriminant Analysis (PLS-MB-DA). PLS-MB-DA was used to determine what predictor variables, (*i.e*. biological determinants, stimulated saliva, resting saliva or microbiota = X blocks) were the most discriminant to characterize the subjects according to variables to be explained (BMI and/or orosensory sensitivity to lipids = Y blocks). (**A**) Comparison between NW (n = 21) and O (n = 17) subjects. (**B**) Comparison between lipid tasters (T, n = 26) and lipid non-tasters (NT, n = 12). (**C**) Comparison between obese tasters (OT, n = 9) and obese non-tasters (ONT, n = 9). Discriminant selection of variables was done using variable importance in the projection (VIP) with a threshold of 1. Mann & Whitney test was used to determine parameters that differed significantly between the three classes (NW vs O, T vs NT and OT vs ONT). Means ± SEM. ^§^P < 0.1; *P < 0.05; **P < 0.01. Amyl, amylase; CA-VI, carbonic anhydrase-VI; Lipo, lipolysis; Lyso, lysozyme; LPS, lipoplolysaccharides; Prot, protein amount; TAC, total anti-oxidant capacity.

According to this multivariate analysis the oral lysozyme activity was found to be the most discriminating variable for the NW group, whereas blood LPS, lipolytic, amylolytic and protein level of resting saliva were prominent in the O subjects (Fig. 6A). The discrete bacterial profile found in the O group, mainly represented by Bifidobacteriaceae and Coriobacteriaceae families, was lacking in NW subjects. Nevertheless, these differences remained either small or not significant (Fig. 6A).

Comparison of T and NT groups has highlighted that NT subjects displayed a higher salivary flow and carbonic anhydrase-VI (CA-VI) activity than T subjects, this last group being mainly characterized by a high lysozyme and TAC activities (Fig. 6B). With respect to microbiota variables, Bacteroidaceae, Prevotellaceae, Sutterellaceae and Sphingomonadaceae families were found to be associated with the NT group while TM7 family and to a lesser extent Lactobacillaceae best characterized the T subjects (Fig. 6B). Therefore, identified differences both in resting and stimulated saliva flow/composition and bacterial families surrounding CVP were found between T and NT groups, suggesting that these parameters might be predictive of changes in oral sensitivity to lipids. By contrast, BMI and waist circumference did not contribute to the discrimination between T and NT phenotypes.

As found in NT subjects regardless of their BMI, ONT subjects were mainly characterized by high salivary flux, CA-VI activity, and to a lesser extent by a set of bacteria families including Sphingomonadaceae, Bacteroidaceae and Prevotellaceae (Fig. 6C). A few significant variables in the T group such as TM7 family and lysozyme activities were also identified in OT groups, but their contribution to the projections were more limited. Surprisingly, variables describing the most ONT subjects were waist circumference and BMI (Fig. 6C). In contrast to T and NT groups, several identified variables were significantly different between OT and ONT as evidenced by the VIP (Fig. 6C), suggesting that ONT subjects constitute a sub-group displaying specific characteristics not shared by all other subjects. Consistent with this assumption, the comparison between NWNT and ONT highlighted the existence of clear differences. The ONT were characterized by CA-VI activity, proteolysis and Shingomonadaceae family mainly, whereas blood LPS levels and Sutterellaceae family were the more significant variables of NWNT group (see Fig. 8, Supplemented data).

## Discussion

Over the last decade, existence of a sixth taste modality responsible for the oral detection of dietary lipids was supported by a growing number of studies both in rodents and humans^17,28^. This finding raises the possibility of functional links between sensitivity for the taste of fat, the preferential consumption of fatty foods, and the obesity risk^4^. We report herein the first analysis on the orosensory sensitivity to lipids in relation to the microenvironment (associated microbiota and saliva) of CVP encompassing most of taste buds found in the lingual epithelium.

Phylogeny of microbiota in the immediate vicinity of CVP, assessed by sequencing the 16S rRNA gene, reveals that the proportion of Proteobacteria was systematically high when compared to what has been commonly described in the fecal matter^29^. At the gene prediction level, the CVP microbiota was also characterized by a broad range of metabolic activities regardless of the group classification of the subjects. Nevertheless, despite identification of a few positive and negative correlations between the frequency of some taxa and BMI, neither the taxonomic profile of the microbiological ecology in direct vicinity of CVP nor the corresponding alpha diversity were sufficient to discriminate clearly between NW and O subjects. Therefore, we analyzed the predicted metagenomes using PICRUSt software, since in human fecal microbiota the metagenomic functional differences are highly and unequivocally associated with the obese phenotype^30^. The gene function analysis of the predicted metagenomes suggested that microbial pathways involved in energy metabolism (*i.e*. amino-acids, lipids, and carbohydrates), as well as modules related to phosphotransferase systems and ribosomal activity, were slightly different between the microbial ecology of CVP from NW and O individuals. Similarly, salivary changes remained limited and insignificant between NW and O subjects. Therefore, no clear association was found between BMI and oral ecology surrounding the CVP. Moreover, BMI appears to be a poor predictor of the fatty taste sensitivity. Consistent with this assumption, a recent meta-analysis concluded to the absence of a correlation between body weight and fatty taste threshold in humans^22^, in contrast to what was found in laboratory rodents^31,32^. This species discrepancy might be explained by the high genetic diversity and the important inter-individual variability of dietary habits in humans.

**Figure 7:**
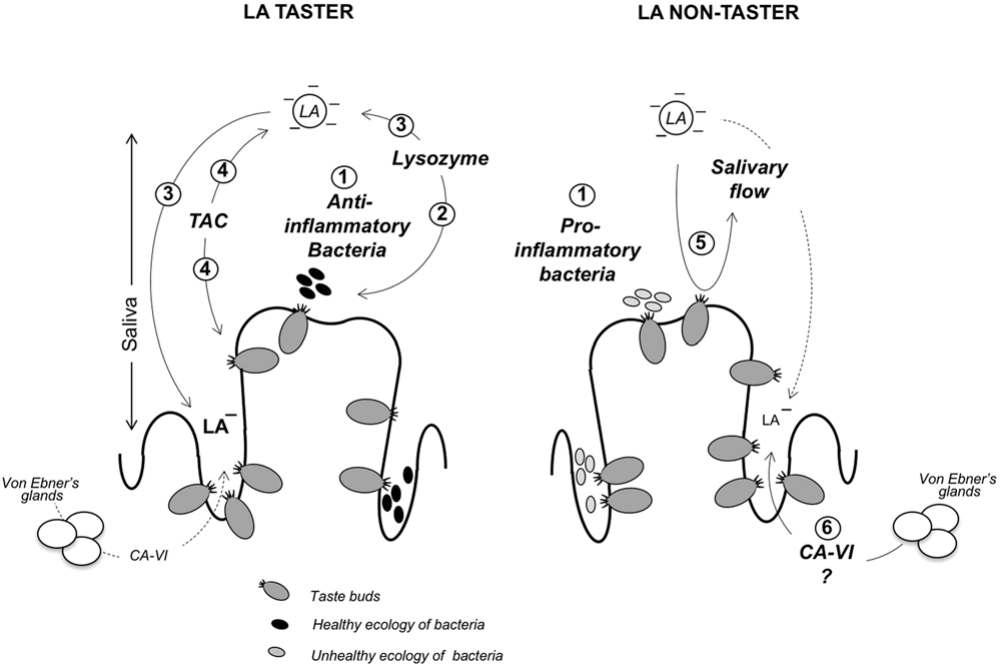
Working model, showing differences in variables constituting the micro-environment surrounding the gustatory circumvallate papillae (CVP) in tasters (T) and non-tasters (NT) and their putative consequence on the fatty taste sensitivity. T are mainly characterized by 1- a host-associated microbiota prone to decrease a local inflammation, 2- a salivary lysozyme activity associated with a lower bacteria diversity, 3- a lysozyme-induced destabilization of the fat emulsion facilitation the release linoleic acid (LA), known to bind and activate lipid sensors in taste buds and 4- a rise in the total antioxidant capacity^37^ of saliva which might facilitate the lipid sensing by protecting unsaturated fatty acids (*e.g*. LA) and gustatory epithelium against lipoperoxidation and tissue damage, respectively. NT are distinguished from T by 5- a microbiota prone to increase a local inflammation, 6- a sustained salivary flux which might decrease the LA access to taste receptor cells and 7- a high carbonic anhydrase-VI (CA-VI) activity whose the modality of action on the oral fat sensitivity to lipids remains elusive.

Therefore, using a similar approach we compared this cohort by targeting the fatty taste sensitivity, independently of BMI. We identified that in the T group the Lactobacillaceae and TM7 families were dominant in the CVP microbiota. The NT subjects were characterized both by an increase in the Bacteroidaceae family and greater bacterial variety, including members of Enterobacteriaceae and Sutterellaceae families, known to be Gram-negative species notably featured by pro-inflammatory LPS molecules. In contrast, increased abundance of the Lactobacillaceae family found in the T subjects display conversely anti-inflammatory functions (Fig. 7-1). Therefore, these data bring the first evidence that resident CVP microbiota can be characterized by a lower capacity for induction of local inflammation in T subjects than in NT individuals. Consistent with a possible role of a local inflammatory environment on sensitivity of fatty taste perception, Gram-negative Porphyromonadaceae and Gram-positive Coriobacteriaceae families were found to be negatively and positively correlated with the orosensitivity to lipids, respectively. Interestingly, taste buds are known to be LPS-sensitive in the mouse. An acute LPS injection (5 mg/kg of body weight) leads to an induction of proinflammatory cytokine production by taste buds and decreases the proliferation of taste receptor cells^26,27^. However, the oro-sensory consequences of such changes remain to be determined. In our cohort, salivary LPS level was not identified as a predictive variable of the oral sensitivity to lipids, since no significant difference was found between T and NT groups. By contrast, our predicted metagenomics data show that numerous microbial metabolic pathways could discriminate between T and NT individuals. They were mainly corresponding to genes involved in the sterol biosynthesis for NT and the biodegradation of organic pollutants (*e.i*. xylene and toluene) in T subjects. Although these dissimilarities remained low and limited, they constitute phenotypic signatures. Analysis of salivary composition also highlighted differences between T and NT groups. We identified that higher lysozyme levels were found in T subjects. This data correlated quite well with the weaker bacterial diversity found in this group (Fig. 7-2) by reason of the well-known antimicrobial activity of this enzyme^33^. Moreover, the lysozyme-mediated destabilization of the negatively charged fatty acid emulsion used in this study has been proposed to increase fatty sensation in the mouth^34,35^ (Fig. 7-3). Interestingly, free fatty acids are responsible for oral fat detection^20^, and the lipid sensor CD36 binds ionized fatty acids with an affinity in the nanomolar range^36^. Finally, the higher total antioxidant capacity^37^ of saliva in T subjects might protect unsaturated FFA (*e.g*. LA) and taste receptors against the lipoperoxidation and the tissue damage, respectively^38^ (Fig. 7-4), facilitating activation of the fatty acid signaling pathway in taste bud cells. Conversely in the NT group, salivary flow rate (Fig. 7-5) and CA-VI activity (Fig. 7-6) were positively correlated to higher LA detection threshold values (*i.e*. lower fatty taste sensitivity). A high salivary flow, diminishing by dilution the LA access to taste receptor cells (*i.e*. CD36), might render these subjects poorly sensitive to lipids. Consistent with this assumption, the perceived intensity and fondness for fat emulsion was found to be related to resting salivary flux in healthy volunteers by using an analogical scale ranking, *i.e*. higher flow correlated with lower perceived intensity and conversely for fondness for the fat emulsion^39^. Although CA-IV activity was identified as a marker of the oral sensitivity to lipids in healthy lean adults^40^, its role on this parameter remains elusive by reason of its multiple functions. This protein, also termed gustin, plays a role both in the salivary buffer capacity^41^, growth and development of taste buds^42^, and bitter taste sensitivity^43^. In brief, these original data strongly suggest that specific microbial and salivary environments surrounding CVP are involved in the fatty taste sensitivity and thereby in the segmentation between T and NT subjects.

Interestingly, obesity amplified the phenotypic differences found between T and NT, suggesting that they are mainly due to obesity once established. We observed a host-associated microbiota shift in favor of bacterial families prone to induce local inflammation (*i.e*. Bacteroidaceae, Sphingomonadaceae and Prevotellaceae) in ONT as compared to OT subjects. Furthermore, ONT subjects were differentiated from OT both by microbial metabolic functions and activities, the predicted abundances for significant differences generally being higher in ONT group. At the salivary level, salivary flow and CA-VI activity, suspected to decrease the fatty taste sensitivity, best defined the ONT group. Finally, ONT displayed a lower body weight and waist circumference than OT, although this unexpected observation must be verified by using a larger cohort. Nevertheless, in obese subjects homozygous for the rs1761667 A allele in the CD36 gene, known to be associated with a reduction of orosensory sensitivity to LCFA^44^, BMI also tends to be higher than in obese FA tasters carrying the G allele^45^. Therefore, these original data have highlighted the existence of an obese subgroup unable to properly perceive orosensory sensations ordinarily triggered by low concentrations of lipids.

In brief, the data reported herein suggest the existence of specific microbiota and salivary signatures in relation with sensitivity of the orosensory detection of dietary lipids. The most relevant data were found when OT were compared to ONT suggesting the existence of an “obese tongue” phenotype in some obese subjects. Since we have previously found using this cohort that ONT preferentially consume energy-dense foods^21^, this finding strongly suggests that the composition of oral microenvironment might be predictive of unhealthy food habits. The current study was not designed to determine whether oral changes observed in ONT were the cause or the consequence of obesity.

In obese patients fecal microbiota is dysbiotic^46^. A reduced bacterial genes richness in feces is often associated with obesity^30^. It causal role and the corresponding mechanism are unknown but are reversed when the proportion of dietary fiber is increased^47^. In rodent models the causality of gut microbiota notably of the ileum mucosal bacteria has been demonstrated to be linked to the reduction of IL17 secreting immune cells from the intestine^48^. In human type 2 diabetes is also featured by a fecal microbiota dysbiosis^49^ which is causal to the disease^50^ and is linked to an increased proportion of LPS releasing bacteria^51^. The endotoxin has been shown to be causal in the inflammatory process of obesity^52^. Therefore, it is tempting to speculate that mechanisms originating from the dysbiotic CVP microbiota, similar to what observed in the intestine, could be leading the obesity phenotype triggering the CVP function towards and impaired sense to lipids. Eventually, based on a multivariate analysis of microbiota, saliva and detection threshold of lipids, this study opens news avenues of research by highlighting associations between parameters usually studied independently. Corroboration of these data by using a larger selection of OT and ONT subjects might lead to validation of easily assayed oral biomarkers associated with this “obese tongue” syndrome and might be useful to identify subgroups of obese subjects who could benefit from an alternative diet.

## Materials and Methods

### Subjects

The subjects were a subgroup of the HumanFATaste study approved by the local ethics committee (Comité de Protection des Personnes Est-1) and registered at Clinical Trials (#NCT02028975). Only volunteers having performed the oral lipid detection threshold test and provided samples of salivary and oral microbiota have been selected. NW (BMI < 25 kg/m^2^, n = 21) and O (BMI ≥ 30 kg/m^2^, n = 17) age-matched, adult, white men participated in the current study. For NW subjects, eligibility criteria were no regular drug intake and plasma triglyceride and glucose levels <1.50 g/L and <6.10 mmol/L, respectively. Because type-2 diabetic subjects have blunted taste responses^53^, obese non-diabetic subjects were selected according to the following inclusion criteria: no hypoglycemic drug intake or surgical treatment of obesity, fasted plasma glucose levels <6.10 mmol/L, and glycated hemoglobin <6.0%. Smokers or former smokers (<3 months) subjects were excluded. Main clinical variables from included subjects are shown on Table 2. All subjects received detailed information about the study and provided a written consent. Participants were subjected to 4 successive sessions. Session 1 included a medical exploration and a sensory screening (European Test of Olfactory Capabilities)^54^. Session 2 was devoted to the threshold determination of linoleic acid (LA). During the session 3 and 4, the oral samplings (i.e. saliva and CVP microbiota) were performed the same day, at the same place (Centre hospitalier universitaire de Dijon, France) and the same hour (*i.e*. 10 a.m., for microbiota samples to avoid any temporal bias).

### Oral LA-threshold detection

The protocol used is fully detailed elsewhere^20,21^. In brief, LA (Sigma Aldrich) oil-in water emulsions were prepared in a solution of 5% acacia gum (wt/wt; Fluka), 5% mineral oil (wt/wt; Cooper), and 0.01% EDTA (wt/wt; VWR international) diluted in evian mineral water (Evian^TM^). Acacia gum and paraffin oil were added to limit viscosity and lubricity differences between control and experimental samples. EDTA was added to prevent the oxidation of LA^20^. Samples were mixed conventionally by using a stirrer (Corning) and homogenized with a sonicator (Misonix sonicator model S-4000; QSonica LLC). The duration of sonication was adapted to LA concentrations to obtain a similar particle size and, thus minimize textural influences. In all cases, sonication was conducted by lapses of 30 s separated by a 1-min pause. Sonication was conducted in a hermetic chamber saturated with nitrogen, and beakers were cooled using an ice bath to limit the formation of oxidized compounds during emulsion preparation. LA-thresholds were determined by using the 3-alternative forced-choice procedure (3-AFC). Participants must identify the sample that is different from the 2 others. Sets were presented in an ascending concentration from 0.00028% to 5% LA (wt/wt) spaced by 0.25 log units (18 solutions in total). The procedure was stopped when the LA sample was correctly identified 3 times, consecutively. This concentration represented the detection-threshold value of the participant. Samples were presented as 5-mL portions in opaque cups and were tested at room temperature. Subjects were instructed to hold the 5 mL solution in their mouth for 7 sec, spit the solution out, and wait for 20 sec before tasting the next sample. The interval between 2 sets was 60–120 sec, during which participants were asked to rinse their mouths with water. Testing was conducted under red lighting and with participants wearing a nose clip to limit visual and olfactory inputs, respectively.

### Oral microbiota samples and analysis

#### Microbiota collection and DNA extraction

Samples from microbiota surrounding the CVP were done in the morning (10 a.m.) in fasted subjects by smear (20 sec) using an oral swab. Total bacterial DNA was extracted, as previously described^55,56^. The protocols for DNA extraction were carefully designed to minimize any risk of contamination between samples or from the experimenters.

#### 16S targeted metagenomics by Miseq sequencing

The bacterial population present in the samples has been determined using next generation high throughput sequencing of variable regions of the 16S rRNA bacterial gene, with a specic protocol established by Vaiomer. The metagenomics workflow was used to classify organisms from a metagenomic sample by amplifying specific regions in the 16S ribosomal RNA gene and was exclusive to bacteria.

#### Library construction and sequencing

The V3-V4 hyper-variable regions of the 16S rDNA gene were amplified from the DNA extracts during a first PCR step using Vaiomer universal 16S primers V2 (Vaiomer 1F 5′-CTTTCCCTA-CACGACGCTCTTCCGATCTTCCTACGGGAGGCAGCAGT-3′ and Vaiomer 2R 5′-GGAGTTCAGACGTGTGCTCTTCCGATCTGGACTACMRGGGTATCTAATCCYKTT-3′) and following the protocol described previously^56^. The joint pair length was set to encompass 476 base pairs amplicon and included specificity for the 16S rDNA gene of 95% of the bacteria in the Ribosomal Database Project. For each sample, a sequencing library was generated by addition of sequencing adapters and multiplexing indexes during a second PCR step as described previously^56^. The pool was denatured, diluted and loaded onto the Illumina MiSeq cartridge according to the manufacturer’s instructions using MiSeq Reagent Kit v3 (2 × 300 bp Paired-End Reads). (Illumina, San Diego, CA, USA).

#### Bioinformatics pipeline

The targeted metagenomic sequences from microbiota were analyzed using the bioinformatics pipeline established by Vaiomer^56^. Briefly, after demultiplexing of the bar coded Illumina paired reads, single read sequences were cleaned and paired for each sample independently into longer fragments. To obtain optimal results, the last 80 bases of the R2 reads were trimmed due to low base quality score. After alignment against a 16S reference database, sequences were clustered into operational taxonomic units^57^ with a 97% identity threshold. Remaining sequencing errors were filtered out by eliminating the OTU with less than 3 sequences, and a taxonomic assignment was performed in order to determine community taxonomic profiles against the RDP database using the RDP Classifier tool. An average of 59,434 raw pairs (118,868 raw reads) per sample were obtained by sequencing and 57,042 pairs (114,084 reads) were conserved after QC filters. To estimate individual CVP microbial alpha diversity, rarefaction curves were generated based on metrics and the number of OTUs present in the samples was determined (Shannon diversity index). The taxonomic output matrix containing the count data for OTUs per sample was processed with the processed with the online Galaxy interface for LEfSe (linear discriminant analysis effect size) algorithm using an alpha parameter significance threshold for the Kruskal-Wallis^58^ test among classes set to 0.05 and the logarithmic LDA score cut-off was set to 2.0. The functional metagenome was inferred from the clustered 16S sequences using the PICRUSt software (version 1.0.0) as per the instructions provided for the Genome Prediction Tutorial for PICRUSt (http://picrust.github.io/picrust/tutorials/genome_prediction.html#genome-prediction-tutorial) with recommended scripts and default settings^25^. As described in the PICRUSt tutorial, the sequences previously grouped into OTU were processed through the QIIME closed reference OTU picking tool with a 97% similarity threshold to obtain a set of OTU IDs from the Greengenes reference collection (gg_otus_13_5.tar.gz) as input for prediction of corresponding metagenomes by PICRUSt. Through this inference process, the abundance values of each OTU were normalized to their respective predicted 16S rRNA copy numbers and then multiplied by the respective gene counts for metagenome prediction. PICRUSt was also used to calculate the nearest sequenced taxon index (NSTI) to quantify dissimilarity between reference genomes and the predicted metagenomes. The resulting core output was a list of Kyoto Encyclopedia of Genes and Genomes (KEGG) orthologues and predicted gene count data for each sample. We used in house scripts to parse the output into KEGG module categories for functional pathways and structural complex hierarchies using the KEGG database (http://www.genome.jp/kegg/module.html). The output matrix containing the relative abundance of KEGG orthologous groups (KO) per sample was processed with the online Galaxy interface for LEfSe using an alpha parameter significance threshold for the Kruskal-Wallis^58^ test among classes set to 0.05 and the logarithmic LDA score cut-off was set to 2.0. Respective cladograms were generated with modules at the lowest level. Quantitative plots of differential features were generated from normalized module level predicted gene data showing means with standard deviation using GraphPad Prism 6 software (GraphPad Software, La Jolla, CA, USA).

### Saliva samples and analysis

Samples of saliva were collected in the morning in fasted subjects subjects. Resting and stimulated saliva were collected in pre weighed cups for 5 min. Stimulated saliva was produced by chewing a piece of Parafilm^59^. Samples were centrifuged (30 min, 15,000 *g*, 4 °C) and the supernatants were stored at −80 °C until biochemical assays. Analyses (flow, protein concentration, enzymatic activities, total antioxidant capacity (TAC) and CA-VI levels were performed as described previously^38,40^. In brief, protein concentration was measured by using the Bradford protein assay (Bio-Rad, France). Proteolytic activity was determined using a Pierce Fluorescent Assay Kit (Pierce Biotechnology, Rockford, IL). Amylolytic activity was assayed using CPNG3 Assay Kit (Biolabo, Maizy, France). Lipolytic activity was explored according a method fully detailed in^38^. Total antioxidant capacity was established using an ORAC Assay kit (CellBioLabs, San Diego, USA). CA-VI was quantified using an Enzyme-Linked ImmunoSorbent Assay kit (USCN Life Science Inc.). Plasma and salivary LPS levels were assayed according to^60^. In brief, LPS-derived 3-hydroxymyristate was extracted from plasma and salivary samples with an organic solvent, separated by reversed phase HPLC, then quantitated by MS/MS.

### Statistical analysis

Statistical analyses (non-parametric Mann-Whitney’s tests and non-parametric Kruskal-Wallis tests followed by Dunn’s multiple comparison tests and Spearman’s correlations) were conducted using the software PRISM v6.05 and the software environment R version 3.3.1. Partial Least Squares-Multiblocs-Discriminant Analysis (PLS-MB-DA) was used to determine which physiological, microbiotal and/or salivary variables (*i.e*. X blocks = predictor variables, Table 1) were the most discriminant to characterize the subjects according their BMI and/or orosensory sensitivity to lipids (*i.e*. Y blocks = variables to be explained). Therefore, three independent analyses were performed to fully explore the differences between the subjects (*i.e*. NW vs O, T vs NT and OT vs ONT). Statistical treatments were performed using the free software R 3.3.0 (http://cran.r-project.org/). The main R package used for multivariate data analyses was ≪pls 2.1-0≫. Statistical treatment requires an pre-processing step as described by Hassani *et al*.^61^. In brief, all the variables (belonging to both X and Y) were mean centred, then variables in X and Y were scaled block-wise to balance the sum of square contribution for different blocks and finally, to explore the systematic variation patterns in X which predicts the systematic variation patterns in Y, PLS algorithm was applied. Relationships between X blocks and Y and weight of each block X for explaining Y were calculated as described elsewhere^62^. Number of components in X that best explain the maximum of variance in Y were chosen based on the percentage of total variance in the Y block explained by the successive components as described previously^63^. In our case study, the components beyond the 2^nd^ dimension explained less than 10%. Therefore, only the outputs of MB-PLS concerning the first two dimensions were interpreted. In order to facilitate interpretation, only X variables that had loadings weights >0.3 were considered. Following PLS-MB-DA approach, discriminant selection of variable was done using variable importance in the projection (VIP) with a threshold of 1 on the first component^63^. Mann & Whitney test was used to determine parameters that differed significantly between the four classes (NW vs O, T vs NT, OT vs ONT and NWT vs NWNT).

**Table 1:** Presentation of the different blocks of variables used in the –MB-PLS-DA analyses.

**Block**	**Abbreviation**	**Definition of the variable**
Biological determinants	BMI	Body Mass Index (kg/m^2^)
	Waist Size	Size of the waist (cm)
	Blood LPS	Blood LPS levels (ng/ml)
	Age	Age of the panellist (years)
Stimulated saliva	Flux_S	Salivary flow of stimulated saliva (ml/min)
	Prot_S	Amount of salivary proteins in stimulated saliva (mg/ml)
	Amyl_S	Amount of Amylase in stimulated saliva (U/ml)
	Lipo_S	Amount of Lipolysis in stimulated saliva (mU/ml)
	Proteo_S	Amount of Proteolysis in stimulated saliva (U/ml)
	Lyso_S	Amount of Lysozyme in stimulated saliva (U/ml)
	TAC_S	Total Antioxydant Capacity of stimulated saliva (µmol/ml)
	CAVI_S	Amount of Carbonic Anhydrase VI in stimulated saliva (ng/ml)
	Cysta_S	Amount of Cystatin SN in stimulated saliva (ng/ml)
	LPSTot_S	Amount of LPS in stimulated saliva (ng/ml)
Resting saliva	Flux_R	Salivary flow of resting saliva (ml/min)
	Prot_R	Amount of salivary proteins in resting saliva (mg/ml)
	Amyl_R	Amount of Amylase in resting saliva (U/ml)
	Lipo_R	Amount of Lipolysis in resting saliva (mU/ml)
	Proteo_R	Amount of Proteolysis in resting saliva (U/ml)
	Lyso_R	Amount of Lysozyme in resting saliva (U/ml)
	TAC_R	Total Antioxydant Capacity of resting saliva (µmol/ml)
	CAVI_R	Amount of Carbonic Anhydrase VI in resting saliva (ng/ml)
	Cysta_R	Amount of Cystatin SN in resting saliva (ng/ml)
Microbiota (Family)	Anaeroplasmataceae	
	Bacteroidaceae	
	Bifidobacteriaceae	
	Coriobacteriaceae	
	Deferribacteraceae	
	Desulfovibrionaceae	
	Eubacteriaceae	
	Helicobacteraceae	
	Lachnospiraceae	
	Lactobacillaceae	
	Porphyromonadaceae	
	Prevotellaceae	
	Rikenellaceae	
	Ruminococcaceae	
	Sphingomonadaceae	
	Sutterellaceae	
	TM7_family_incertae_sedis	
	unclassified	

It is noteworthy that the number of subjects in which the oral microbiota was sequenced (n = 45) and in which salivary variables were analyzed (n = 38) differs. In some subjects, the salivary analyses failed to provide results. We however performed statistical analyses with 45 and 38 subjects, but could not found significant differences suggesting that the missing subjects had no influence on the statistics.

## Electronic supplementary material

Dataset 1
