# Author Correction: Obese Subjects With Specific Gustatory Papillae Microbiota and Salivary Cues Display an Impairment to Sense Lipids

**DOI:** 10.1038/s41598-018-27701-w

**Published:** 2018-06-22

**Authors:** Philippe Besnard, Jeffrey E. Christensen, Hélène Brignot, Arnaud Bernard, Patricia Passilly-Degrace, Sophie Nicklaus, Jean-Paul Pais de Barros, Xavier Collet, Benjamin Lelouvier, Florence Servant, Vincent Blasco-Baque, Bruno Verges, Laurent Lagrost, Gilles Feron, Rémy Burcelin

**Affiliations:** 1 UMR Lipides/Nutrition/Cancer U1231 INSERM/Univ Bourgogne-Franche Comté/AgroSupDijon, 21000 Dijon, France; 2 I2MC Institut des maladies métaboliques et cardiovasculaires/UMR 1048 INSERM/Univ Toulouse III Paul Sabatier, 31400 Toulouse, France; 3 Centre des Sciences du Goût et de l’Alimentation, AgroSup Dijon, CNRS, INRA, Univ. Bourgogne Franche-Comté, F-21000 Dijon, France; 4 Vaiomer S.A.S, 31670 Labège, France

Correction to: *Scientific Reports* 10.1038/s41598-018-24619-1, published online 30 April 2018

The original version of this Article contained an error in the spelling of the author Vincent Blasco-Baque, which was incorrectly given as Vincent Blasco. This has now been corrected in the PDF and HTML versions of the Article, as well as the accompanying Supplementary Information file.

