# Supplementary material for: Obese Subjects With Specific Gustatory Papillae Microbiota and Salivary Cues Display an Impairment to Sense Lipids
Source: Sci Rep. 2018 Apr 30;8:6742. doi: 10.1038/s41598-018-24619-1 (PMC5928223; doi:10.1038/s41598-018-24619-1)
Supplement: Supplementary file 1 — Dataset 1 [file 41598_2018_24619_MOESM1_ESM.docx]

**Obese Subjects With Specific Gustatory Papillae Microbiota And Salivary Cues Display An Impairment To Sense Lipids**

Philippe Besnard^1^*, Jeffrey E. Christensen^2^, Hélène Brignot^3^, Arnaud Bernard^1^, Patricia Passilly-Degrace^1^, Sophie Nicklaus^3,^ Jean-Paul Pais de Barros^1^, Xavier Collet^2^, Benjamin Lelouvier^4^, Florence Servant^4^, Vincent Blasco-Baque^2^, Bruno Verges^1^, Laurent Lagrost^1^, Gilles Féron^3^, Rémy Burcelin^2^*

^1^ UMR Lipides/Nutrition/Cancer U1231 INSERM/Univ Bourgogne-Franche Comté/ AgroSupDijon, 21000 Dijon, France

^2^ UMR 1048 INSERM/Univ Toulouse III Paul Sabatier, 31000 Toulouse, France

^3^ Centre des Sciences du Goût et de l'Alimentation, AgroSup Dijon, CNRS, INRA, Univ. Bourgogne Franche-Comté, F-21000 Dijon, France.

^4^ Vaiomer S.A.S, 31670 Labège, France

* Corresponding authors

P. Besnard, NUTox AgroSupDijon, 1 Esplanade Erasme 21000 Dijon, France

Tel +33 (0)380 774 091 ; [pbesnard@u-bourgogne.fr](mailto:pbesnard@u-bourgogne.fr)

R. Burcelin Institut des Maladies Métaboliques et Cardiovasculaire INSERM1048, Hôpital Rangueil, 31400, Toulouse France

Tel +33 (0)561 325 621; [remy.burcelin@inserm.fr](mailto:remy.burcelin@inserm.fr)

**Supplementary Information Contents**

Supplementary Tables 1 – 2

Supplementary Figures 1

**Table 1**: Analysis of four blocks of predictors (*i.e.* selected biological determinants. resting saliva. stimulated saliva and microbiota composition) aggregating 42 variables in function of BMI (NW vs O) or fatty taste sensitivity (T *vs* NT and OT *vs* ONT).

|  | **Obese (O)** | | **Normal Weight (NW)** | | **Taster (T)** | | **Non Taster (NT)** | | **Obese Taster (OT)** | | **Obese Non Taster (ONT)** | |
| --- | --- | --- | --- | --- | --- | --- | --- | --- | --- | --- | --- | --- |
|  | **n=17** | | **n=21** | | **n=26** | | **n=12** | | **n=9** | | **n=8** | |
|  | **Mean** | ***SD*** | **Mean** | ***SD*** | **Mean** | ***SD*** | **Mean** | ***SD*** | **Mean** | ***SD*** | **Mean** | ***SD*** |
| **Physiological Variables** |  |  |  |  |  |  |  |  |  |  |  |  |
| BMI | *34.5* | *3.51* | *22.3* | *1.7* | *27.2* | *7.13* | *29* | *5.69* | *36.3* | *3.02* | *32.5* | *2.97* |
| Waist circumference (m) | *1.15* | *0.1* | *0.86* | *0.09* | *0.98* | *0.19* | *1.01* | *0.12* | *1.21* | *0.07* | *1.08* | *0.06* |
| LA detection threshold (% w/w) | *2.24* | *3.89* | *0.37* | *0.74* | *0.04* | *0.07* | *3.72* | *3.99* | *0.03* | *0.03* | *4.72* | *4.61* |
| Blood LPS (ng/ml) | *27.2* | *7.92* | *24.8* | *4.32* | *25.5* | *6.84* | *26.8* | *4.74* | *28.6* | *9.84* | *25.7* | *5.23* |
| Age | *52.4* | *11.5* | *51.5* | *14.6* | *50.9* | *13.5* | *54.1* | *12.5* | *50.8* | *9.18* | *54.1* | *14.1* |
| **Stimulated Saliva** |  |  |  |  |  |  |  |  |  |  |  |  |
| Flux (ml) | *0.47* | *0.21* | *0.57* | *0.28* | *0.48* | *0.24* | *0.64* | *0.26* | *0.38* | *0.13* | *0.57* | *0.24* |
| Prot (mg/ml) | *0.56* | *0.19* | *0.5* | *0.19* | *0.55* | *0.21* | *0.49* | *0.14* | *0.60* | *0.21* | *0.52* | *0.15* |
| Amyl (UI/ml) | *674* | *326* | *1411* | *3051* | *1290* | *2741* | *629* | *373* | *712* | *275* | *633* | *391* |
| Lipo (mUI/ml) | *1.75* | *1.48* | *1.37* | *0.88* | *1.59* | *1.22* | *1.42* | *1.16* | *2.17* | *1.57* | *1.27* | *1.31* |
| Proteo (UI/ml) | *349* | *340* | *285* | *260* | *300* | *270* | *345* | *355* | *253* | *275* | *457* | *390* |
| Lyso (U/ml) | *148* | *103* | *201* | *170* | *197* | *166* | *134* | *70.1* | *178* | *126* | *114* | *59.5* |
| TAC (µmol/ml) | *792* | *470* | *759* | *403* | *839* | *481* | *633* | *245* | *920* | *573* | *648* | *291* |
| CA-VI (ng/ml) | *5.47* | *3.05* | *5.78* | *4.77* | *5.6* | *4.27* | *5.74* | *3.66* | *3.91* | *2.27* | *7.22* | *2.96* |
| Cysta (ng/ml) | *210* | *152* | *189* | *148* | *197* | *145* | *201* | *162* | *235* | *188* | *182* | *105* |
| Total LPS (ng/ml) | *46.4* | *28.8* | *48.1* | *34.4* | *50.2* | *34.2* | *41.1* | *25.5* | *50.8* | *32.6* | *41.5* | *25.2* |
| **Resting Saliva** |  |  |  |  |  |  |  |  |  |  |  |  |
| Flux (ml) | *0.15* | *0.07* | *0.14* | *0.09* | *0.12* | *0.07* | *0.19* | *0.09* | *0.11* | *0.06* | *0.18* | *0.06* |
| Prot (ml) | *1.26* | *0.74* | *0.99* | *0.41* | *1.09* | *0.56* | *1.15* | *0.67* | *1.20* | *0.79* | *1.34* | *0.73* |
| Amyl (UI/ml) | *2202* | *2914* | *1320* | *1723* | *1454* | *1799* | *2279* | *3247* | *1405* | *1733* | *3098* | *3773* |
| Lipo (mUI/ml) | *4.06* | *5.3* | *2.14* | *1.68* | *2.8* | *3.49* | *3.41* | *4.61* | *4.0* | *5.43* | *4.12* | *5.52* |
| Proteo (UI/ml) | *472* | *384* | *594* | *754* | *571* | *692* | *472* | *407* | *325* | *320* | *637* | *401* |
| Lyso (U/ml) | *479* | *281* | *630* | *321* | *630* | *320* | *417* | *233* | *545* | *338* | *405* | *193* |
| TAC (µmol/ml) | *1532* | *842* | *1558* | *684* | *1645* | *778* | *1333* | *658* | *1625* | *950* | *1426* | *752* |
| CA-VI ng/ml) | *5.21* | *3.35* | *5.15* | *3.14* | *4.62* | *3.1* | *6.39* | *3.18* | *3.9* | *3.27* | *6.71* | *2.94* |
| Cysta (ng/ml) | *745* | *592* | *611* | *561* | *647* | *582* | *724* | *570* | *698* | *576* | *798* | *644* |
| **Microbiota (Family - %)** |  |  |  |  |  |  |  |  |  |  |  |  |
| Anaeroplasmataceae | *0.25* | *0.3* | *0.18* | *0.33* | *0.19* | *0.31* | *0.26* | *0.35* | *0.20* | *0.20* | *0.31* | *0.39* |
| Bacteroidaceae | *3.35* | *5.9* | *2.46* | *2.12* | *1.94* | *1.93* | *4.86* | *6.69* | *0.90* | *0.67* | *6.10* | *7.92* |
| Bifidobacteriaceae | *0.3* | *0.53* | *0.08* | *0.13* | *0.12* | *0.32* | *0.3* | *0.48* | *0.23* | *0.52* | *0.39* | *0.57* |
| Coriobacteriaceae | *0.35* | *0.21* | *0.29* | *0.15* | *0.31* | *0.18* | *0.33* | *0.18* | *0.37* | *0.21* | *0.33* | *0.21* |
| Deferribacteraceae | *0.09* | *0.19* | *0.16* | *0.38* | *0.16* | *0.37* | *0.06* | *0.13* | *0.15* | *0.25* | *0.02* | *0.03* |
| Desulfovibrionaceae | *0.84* | *0.91* | *1.44* | *2.02* | *0.97* | *1.58* | *1.61* | *1.72* | *0.73* | *1.01* | *0.96* | *0.82* |
| Eubacteriaceae | *0.01* | *0.02* | *0.01* | *0.02* | *0.01* | *0.02* | *0.01* | *0.03* | *0.01* | *0.02* | *0.00* | *0.01* |
| Helicobacteraceae | *4.59* | *4.18* | *4.54* | *4.33* | *5.12* | *4.41* | *3.36* | *3.6* | *5.30* | *4.41* | *3.78* | *4.03* |
| Lachnospiraceae | *42.9* | *9.66* | *44* | *11.4* | *42.7* | *9.78* | *45.3* | *12.2* | *41.0* | *2.97* | *45.1* | *13.9* |
| Lactobacillaceae | *3.57* | *3.59* | *4.37* | *3.91* | *4.58* | *4.01* | *2.77* | *2.86* | *4.09* | *4.15* | *2.98* | *3.00* |
| Porphyromonadaceae | *23* | *6.98* | *22.5* | *6.23* | *23.1* | *6.14* | *22* | *7.42* | *25.1* | *4.77* | *20.6* | *8.58* |
| Prevotellaceae | *2.55* | *2.21* | *2.35* | *2.28* | *2.11* | *1.66* | *3.15* | *3.09* | *1.70* | *1.08* | *3.51* | *2.81* |
| Rikenellaceae | *2.52* | *2.57* | *2.39* | *1.65* | *2.6* | *2.41* | *2.13* | *1.1* | *2.89* | *3.38* | *2.11* | *1.29* |
| Ruminococcaceae | *6.67* | *1.81* | *6.91* | *2.23* | *7.11* | *2.08* | *6.13* | *1.8* | *6.85* | *1.76* | *6.47* | *1.98* |
| Sphingomonadaceae | *0.01* | *0.01* | *0.01* | *0.02* | *0.01* | *0.01* | *0.02* | *0.03* | *0.00* | *0.01* | *0.02* | *0.02* |
| Sutterellaceae | *0.46* | *0.59* | *0.37* | *0.33* | *0.32* | *0.29* | *0.62* | *0.67* | *0.36* | *0.28* | *0.58* | *0.82* |
| TM7_family_incertae_sedis | *0.17* | *0.24* | *0.38* | *0.53* | *0.38* | *0.49* | *0.07* | *0.16* | *0.22* | *0.28* | *0.10* | *0.19* |
| unclassified | *7.45* | *4.37* | *6.98* | *4.06* | *7.41* | *4.02* | *6.73* | *4.56* | *8.54* | *3.60* | *6.23* | *5.07* |

| **ID** | **Age** | **Group** | **BMI** | **Waist circumference (m)** | **Fasting glycemia (mmol/l)** | **Total Chol (mmol/l)** | **LDL (mmol/l)** | **HDL (mmol/l)** | **TG (mmol/l)** | **HbA1c (%)** | **LA detection threshold (% w/w)** | **Taster** |
| --- | --- | --- | --- | --- | --- | --- | --- | --- | --- | --- | --- | --- |
| *3* | *76* | *Lean* | *28.2* | *0.98* | *4.70* | *3.81* | *1.98* | *1.57* | *0.58* | *5.6* | *1.5811* | *N* |
| *4* | *45* | *Obese* | *36.3* | *1.20* | *5.14* | *4.67* | *2.59* | *1.12* | *2.11* | *5.2* | *0.00889* | *Y* |
| *6* | *54* | *Obese* | *39.1* | *1.20* | *5.42* | *4.99* | *2.96* | *1.41* | *1.37* | *5.4* | *0.05* | *Y* |
| *7* | *35* | *Obese* | *34.0* | *1.10* | *4.38* | *5.47* | *3.65* | *1.26* | *1.24* | *4.5* | *0.0158* | *Y* |
| *8* | *61* | *Obese* | *33.5* | *1.13* | *5.38* | *4.06* | *2.32* | *1.03* | *1.57* | *5.8* | *0.5* | *N* |
| *9* | *62* | *Lean* | *19.8* | *0.72* | *4.80* | *4.12* | *1.94* | *1.83* | *0.77* | *5.3* | *0.0281* | *Y* |
| *10* | *69* | *Lean* | *24.0* | *0.94* | *5.91* | *6.23* | *4.01* | *1.89* | *0.72* | *5.5* | *0.005* | *Y* |
| *12* | *69* | *Obese* | *32.2* | *1.04* | *5.97* | *6.32* | *4.50* | *1.07* | *1.64* | *5.8* | *0.8891* | *N* |
| *14* | *63* | *Lean* | *21.1* | *0.95* | *5.59* | *4.41* | *2.64* | *1.17* | *1.31* | *5.1* | *0.00889* | *Y* |
| *15* | *50* | *Obese* | *32.8* | *1.13* | *6.08* | *6.63* | *2.88* | *0.95* | *7.71* | *5.8* | *0.0281* | *Y* |
| *17* | *52* | *Lean* | *22.8* | *0.83* | *4.73* | *5.95* | *3.39* | *2.03* | *1.17* | *5.0* | *0.00889* | *Y* |
| *20* | *63* | *Obese* | *31.6* | *1.02* | *5.59* | *5.46* | *3.62* | *1.32* | *1.15* | *5.7* | *0.5* | *N* |
| *21* | *29* | *Lean* | *22.0* | *0.80* | *4.80* | *5.56* | *3.76* | *1.33* | *1.04* | *5.5* | *0.8891* | *N* |
| *22* | *62* | *Lean* | *24.0* | *0.84* | *4.56* | *5.64* | *3.40* | *1.54* | *1.55* | *4.9* | *0.2811* | *Y* |
| *23* | *49* | *Obese* | *38.7* | *1.18* | *5.78* | *5.32* | *3.34* | *0.92* | *2.33* | *6.0* | *0.00158* | *Y* |
| *24* | *58* | *Lean* | *22.2* | *0.91* | *5.27* | *5.69* | *3.52* | *1.76* | *0.90* | *5.9* | *0.01581* | *Y* |
| *26* | *20* | *Lean* | *24.0* | *0.75* | *4.82* | *3.95* | *2.42* | *1.17* | *0.80* | *5.1* | *0.2811* | *Y* |
| *29* | *61* | *Obese* | *38.5* | *1.31* | *4.62* | *5.84* | *3.27* | *1.80* | *1.69* | *5.4* | *0.05* | *Y* |
| *30* | *65* | *Lean* | *22.6* | *0.92* | *5.41* | *7.26* | *4.68* | *2.13* | *1.00* | *5.4* | *0.0889* | *Y* |
| *31* | *47* | *Lean* | *19.7* | *0.75* | *6.19* | *5.28* | *3.45* | *1.41* | *0.93* | *5.9* | *0.015* | *Y* |
| *32* | *64* | *Lean* | *20.8* | *1.00* | *4.67* | *5.51* | *3.55* | *1.59* | *0.81* | *5.8* | *1.581* | *N* |
| *33* | *63* | *Lean* | *20.9* | *0.81* | *5.18* | *6.29* | *3.68* | *2.30* | *0.69* | *5.3* | *0.0005* | *Y* |
| *34* | *64* | *Lean* | *24.1* | *0.93* | *4.66* | *6.05* | *3.73* | *1.98* | *0.75* | *5.3* | *0.015* | *Y* |
| *35* | *46* | *Obese* | *33.3* | *1.11* | *5.52* | *6.10* | *4.01* | *1.15* | *2.06* | *5.7* | *<10* | *N* |
| *38* | *23* | *Lean* | *24.4* | *0.82* | *4.98* | *4.30* | *2.45* | *1.52* | *0.72* | *5.2* | *0.0281* | *Y* |
| *39* | *35* | *Lean* | *25.2* | *0.80* | *4.84* | *5.77* | *3.52* | *1.74* | *1.12* | *5.3* | *0.00281* | *Y* |
| *43* | *64* | *Lean* | *19.7* | *0.77* | *5.12* | *6.02* | *3.14* | *2.41* | *1.03* | *5.9* | *0.05* | *Y* |
| *44* | *47* | *Obese* | *29.8* | *0.99* | *5.11* | *6.34* | *3.97* | *1.04* | *4.31* | *5.4* | *5* | *N* |
| *45* | *43* | *Lean* | *20.2* | *0.99* | *4.00* | *5.39* | *3.04* | *2.04* | *0.68* | *5.3* | *0.00889* | *Y* |
| *48* | *54* | *Lean* | *22.9* | *0.98* | *3.96* | *6.61* | *4.28* | *1.88* | *0.99* | *5.4* | *2.812* | *N* |
| *49* | *35* | *Lean* | *21.6* | *0.78* | *4.26* | *4.55* | *2.59* | *1.72* | *0.52* | *5.0* | *0.0281* | *Y* |
| *50* | *40* | *Lean* | *23.9* | *0.92* | *NA* | *5.17* | *3.58* | *1.02* | *1.26* | *5.5* | *0.005* | *Y* |
| *52* | *59* | *Obese* | *31.6* | *1.32* | *NA* | *5.54* | *3.62* | *1.24* | *1.50* | *NA* | *0.005* | *Y* |
| *55* | *63* | *Obese* | *30.9* | *1.13* | *5.59* | *7.22* | *4.52* | *2.00* | *1.55* | *5.5* | *0.8891* | *N* |
| *56* | *26* | *Obese* | *38.9* | *1.13* | *NA* | *NA* | *NA* | *NA* | *NA* | *NA* | *<10* | *N* |
| *72* | *58* | *Obese* | *29.6* | *1.06* | *5.19* | *5.20* | *2.85* | *1.94* | *0.90* | *5.6* | *<10* | *N* |
| *73* | *61* | *Obese* | *35.7* | *1.24* | *6.06* | *6.39* | *3.71* | *2.00* | *1.50* | *6.0* | *0.0889* | *Y* |
| *79* | *43* | *Obese* | *40.1* | *1.24* | *4.88* | *5.03* | *3.06* | *1.33* | *1.40* | *5.0* | *0.0158* | *Y* |

**Table 2**(supplemented data) : Main characteristics of subjects included in the study


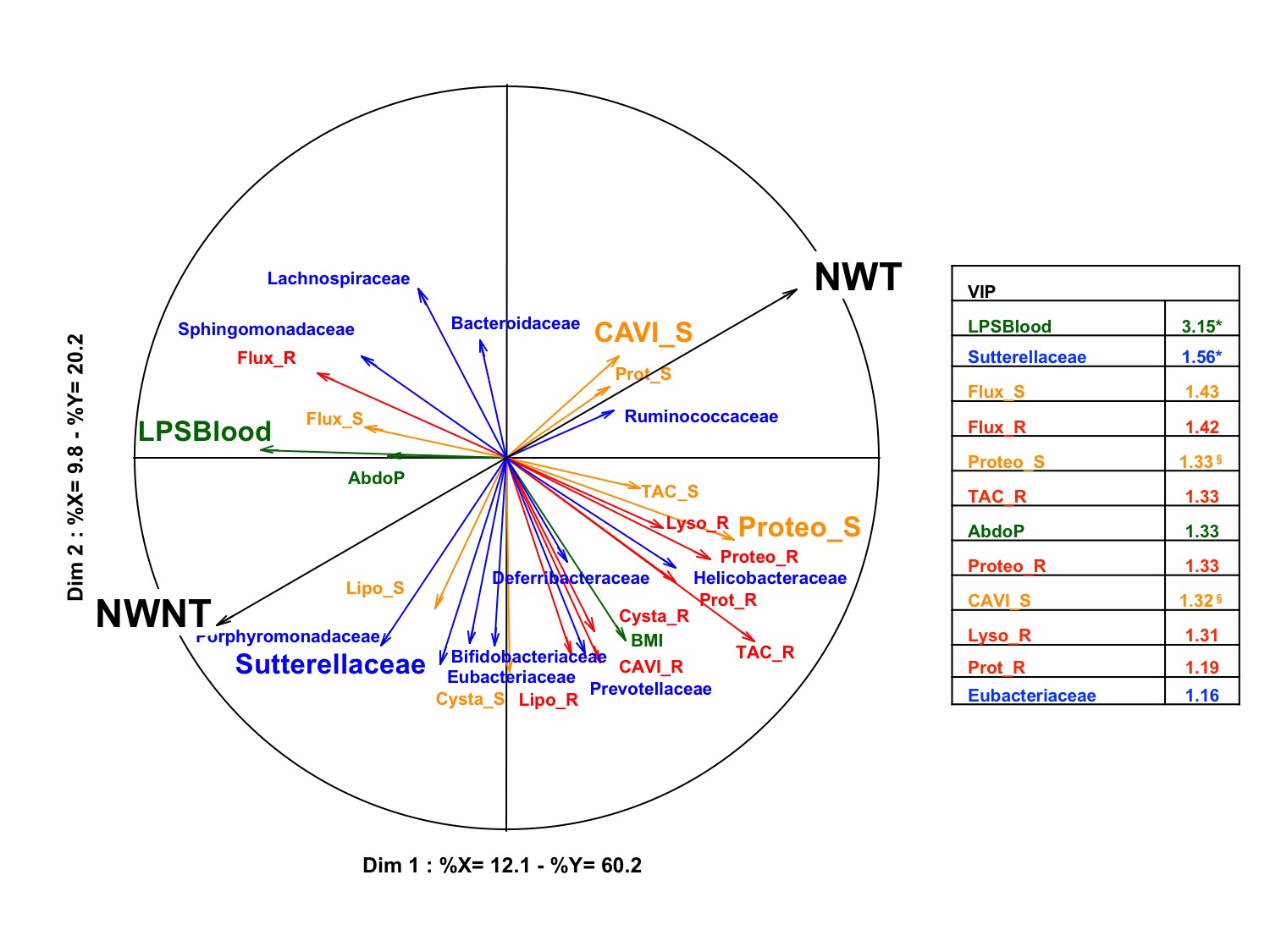


**Fig. S1: supplemented data: Partial Least Squares-Multiblocs-Discriminant Analysis (PLS-MB-DA) in NWT and NWNT.** PLS-MB-DA was used to determine what predictor variables, (*i.e.* biological determinants, stimulated saliva, resting saliva or microbiota = X blocks) were the most discriminant to characterize the subjects according to variables to be explained (orosensory sensitivity to lipids = Y blocks). Comparison between normal-weight tasters (NWT, n= 4) and normal-weight non-tasters (NWNT, n= 17). Discriminant selection of variables was done using variable importance in the projection (VIP) with a threshold of 1. Mann & Whitney test was used to determine parameters that differed significantly between NWT & NWNT.

Means± SEM. §, P<0.1; *, P<0.05. Amyl, amylase; CA-IV, carbonic anhydrase; Lipo, lipolysis; Lyso, lysozyme; LPS, lipopolysaccharides; Prot, protein amount; TAC, total anti-oxidant capacity.
